# Supplementary material for: Enabling AI in synthetic biology through Construction File specification
Source: PLoS One. 2023 Nov 13;18(11):e0294469. doi: 10.1371/journal.pone.0294469 (PMC10642840; doi:10.1371/journal.pone.0294469)
Supplement: S1 File — This ZIP file contains all essential supporting documents, including specification documents, chats, movies, Construction Files, and sequences, as referenced in the main text. A detailed description of contents is provided in the accompanying README.txt. (ZIP) [file pone.0294469.s001.zip › S1_CF_Supplementa/Chats/cf_to_pydna.html]

CF to pydna - A ShareGPT conversation 

Model: GPT-4

# CF Shorthand Specification
## Layer 0: Basic Concept
A Construction File (CF) is a list of Steps, with each Step representing a specific operation in a molecular biology experiment. Each Step is written on a new line. Parameters are separate by whitespace, preferably TSV. The Step includes the names of input DNA sequence(s), non-sequence parameters, and ends with the name of the product DNA sequence. The input sequences can refer to products of previous steps. The product is the output of the operation.
In addition, a CF can include sequences in the form of 'name sequence' lines.
Comment lines follow '#', '//', or '/\*comment\*/' syntax.
## Layer 1: Core Operations
This layer defines a core set of operations: PCR, Digest, Ligate, GoldenGate, Gibson, and Transform. Each operation has specific parameters and (optionally) a product name.
## Examples
- `PCR ForwardPrimer ReversePrimer Template ProductName`
- ForwardPrimer, ReversePrimer, Template: Names of DNA sequences
- `Digest DNA Enzymes FragmentSelection ProductName`
- DNA: Name of the DNA sequence to be digested
- Enzymes: List of enzymes to be used, e.g. EcoRI,BamHI
- FragmentSelection: Index indicating the chosen fragment post-digestion, according to orientation and origin given in the input DNA.
- `Ligate Fragment1 Fragment2 ProductName`
- Fragment1, Fragment2: Names of DNA sequences
- `GoldenGate Fragment1 Fragment2 Enzyme ProductName`
- Fragment1, Fragment2: Names of DNA sequences
- Enzyme: Type IIS-like enzyme to be used along with ligase. e.g. BsaI
- `Gibson Fragment1 Fragment2 ProductName`
- Fragment1, Fragment2: Names of DNA sequences
- `Transform Plasmid Host Antibiotic Temperature ProductName`
- Plasmid: Name of the DNA sequence
- Host: Bacterial strain
- Antibiotics: Antibiotics used, e.g. Amp,Kan
- Temperature: Incubation temperature in Celsius, e.g. 37
Here is an example I wish to discuss:
//Figure 1 Example: PCR, cut, and ligate with palindromic type II six-cutters of two fragments
//Derived from https://openwetware.org/wiki/Arking:JCAOligoTutorial1
# Cloning Steps
PCR ca1067F ca1067R pSB1AK3-b0015 pcrpdt
Digest pcrpdt EcoRI,SpeI 1 pcrdig
Digest pSB1A2-I13521 EcoRI,SpeI 1 vectdig
Ligate pcrdig vectdig lig
Transform lig DH10B Amp 37 pSB1A2-Bca9128

The example you provided demonstrates a complete molecular biology experiment in a Construction File (CF) format. It can be dissected step-by-step:

1. `PCR ca1067F ca1067R pSB1AK3-b0015 pcrpdt`: This line means a PCR reaction is carried out using primers `ca1067F` and `ca1067R` on the template DNA sequence `pSB1AK3-b0015`. The resultant product is named `pcrpdt`.
2. `Digest pcrpdt EcoRI,SpeI 1 pcrdig`: This command indicates a DNA digestion step where the DNA product from the previous PCR step (`pcrpdt`) is digested by the enzymes `EcoRI` and `SpeI`. The first fragment (as specified by `1`) resulting from this digestion is selected and named `pcrdig`.
3. `Digest pSB1A2-I13521 EcoRI,SpeI 1 vectdig`: This step is similar to the previous one but performed on a different DNA sequence named `pSB1A2-I13521`. The selected fragment is named `vectdig`.
4. `Ligate pcrdig vectdig lig`: This command represents a ligation process where the two fragments `pcrdig` and `vectdig` from the previous digestion steps are ligated together. The product from this ligation step is named `lig`.
5. `Transform lig DH10B Amp 37 pSB1A2-Bca9128`: The last command is the transformation of the plasmid `lig` into `DH10B` bacterial strain. The transformation culture is then subjected to antibiotic selection (`Amp`) at `37` degrees Celsius. The resulting transformed strain is named `pSB1A2-Bca9128`.

This CF format serves as a precise and succinct way to outline molecular biology experiments, allowing for clear communication and interpretation of experimental design and steps.

Write a pydna script I can run in Google Colab to simulate this CF

Sure, here's a Python script using the `pydna` library that you can run in Google Colab to simulate the Construction File (CF) you've provided.

Note that the actual sequences for primers, templates, and plasmids aren't provided, so I've added placeholders. In your actual script, you would replace these with actual DNA sequences.

```
pythonCopy code


# Import necessary libraries
from pydna.dseq import Dseq
from pydna.amplify import pcr
from pydna.assembly import Assembly
from pydna.readers import read
from pydna.genbank import Genbank

# Install Biopython if not done already
!pip install biopython pydna

# Connect to Genbank
gb = Genbank("YourEmailAddress@example.com")  # Replace with your email

# Define sequences (replace "ATG..." with actual sequences)
ca1067F = Dseq("ATG...")
ca1067R = Dseq("ATG...")
pSB1AK3_b0015 = gb.nucleotide("Part:BBa_B0015")  # Fetch sequence from Genbank

# PCR Step
pcrpdt = pcr(ca1067F, ca1067R, pSB1AK3_b0015)

# Define enzymes
from Bio.Restriction import EcoRI, SpeI
enzymes = [EcoRI, SpeI]

# Digest Steps
pcrdig = pcrpdt.cut(enzymes)[0]  # Assuming fragment of interest is the first one

pSB1A2_I13521 = gb.nucleotide("Part:BBa_I13521")  # Fetch sequence from Genbank
vectdig = pSB1A2_I13521.cut(enzymes)[0]  # Assuming fragment of interest is the first one

# Ligation Step
assembly = Assembly([pcrdig, vectdig])
lig = assembly.circular_products()[0]

# Write output sequences to files
with open("pcrpdt.gb", "w+") as file:
    file.write(pcrpdt.format("genbank"))

with open("lig.gb", "w+") as file:
    file.write(lig.format("genbank"))
```

This script provides a simulation for the steps in the CF but doesn't fully cover the `Transform` operation as the biological transformation process is not straightforward to simulate in a script (i.e., it involves biological processes like transformation efficiency, antibiotic selection, etc., that cannot be accurately modeled). However, the output files `pcrpdt.gb` and `lig.gb` are the desired end products of your genetic manipulation, representing the result of transformation.
