# Supplementary material for: Enabling AI in synthetic biology through Construction File specification
Source: PLoS One. 2023 Nov 13;18(11):e0294469. doi: 10.1371/journal.pone.0294469 (PMC10642840; doi:10.1371/journal.pone.0294469)
Supplement: S1 File — This ZIP file contains all essential supporting documents, including specification documents, chats, movies, Construction Files, and sequences, as referenced in the main text. A detailed description of contents is provided in the accompanying README.txt. (ZIP) [file pone.0294469.s001.zip › S1_CF_Supplementa/Chats/java_simulator_conversion.html]

I need to write a function in google apps
script for : function gibson(dnaSequences)
{ } It inputs an array of strings representing dna
sequences with homology arms between them. It returns the product of reacting
via the gibson isothermal assembly method

ChatGPT

The Gibson Assembly is a method for assembling
multiple DNA fragments into a single, contiguous DNA molecule. Here's a sample
implementation in Google Apps Script:

scssCopy code

function gibson(dnaSequences) { // Define the homology arms var homologyArms = getHomologyArms(dnaSequences); // Perform the Gibson Assembly reaction var
product = performGibsonAssembly(homologyArms);
// Return the assembled DNA molecule return product; } function getHomologyArms(dnaSequences) { //
Code to extract the homology arms from the input DNA sequences } function performGibsonAssembly(homologyArms)
{ // Code to perform the Gibson Assembly reaction using the homology arms }

Note that the **getHomologyArms**
and **performGibsonAssembly** functions would need
to be implemented based on the specific requirements of the Gibson Assembly
reaction being performed.

OK, implement getHomologyArms

ChatGPT

Sure, here's a sample implementation for the **getHomologyArms** function:

javascriptCopy code

function getHomologyArms(dnaSequences) { var homologyArms
= []; // Loop through each DNA sequence for (var i = 0;
i < dnaSequences.length;
i++) { var sequence = dnaSequences[i]; // Extract the homology arm from the current DNA
sequence var homologyStart = sequence.indexOf("homology");
var homologyEnd = homologyStart
+ "homology".length; var homologyArm = sequence.substring(homologyStart, homologyEnd); //
Add the extracted homology arm to the array homologyArms.push(homologyArm); } return homologyArms;
}

In this implementation, we loop through each
DNA sequence in the input array and use the **indexOf**
method to find the starting index of the word "homology". The length
of the word "homology" is then used to determine the ending index,
and the substring between these two indices is extracted as the homology arm.
The extracted homology arm is then added to the **homologyArms**
array, which is returned at the end of the function.

Note that this implementation assumes that the
word "homology" is present in each DNA sequence and that it
represents the start of the homology arm. If this is not the case, the
implementation would need to be adjusted accordingly.

start over on this. The signature should stay
the same, but I want to base it on this code: private Polynucleotide simGibson(List<Polynucleotide> assemblyFragments)
throws Exception { RevComp revComp
= new RevComp(); revComp.initiate();
while (assemblyFragments.size() > 1) {
Polynucleotide currFrag = assemblyFragments.remove(0);
int currLen = currFrag.getSequence().length();
//\* declared currLen outside of loop String homologyRegion = currFrag.getSequence().substring(currLen - 20); //can now use currLen
Polynucleotide matchedFrag = null; int currHomologousRegionStartIndex; // The index of the
beginning of the homologous region on the current strand int matchedHomologousRegionStartIndex; // The index of the
beginning of the homologous region on the matched strand int matchedHomologousRegionEndIndex = 0; // Index on matchedFrag of the end of the homologous region (including
20 bp) for (Polynucleotide tempFrag : assemblyFragments) { //int currLen
= currFrag.getSequence().length(); moved this outside
of the for loop // If there is a match between the last 20 bp of currFrag and the forward strand of tempFrag
if (tempFrag.getSequence().contains(homologyRegion)) { matchedFrag = tempFrag; matchedHomologousRegionEndIndex
= 20 + tempFrag.getSequence().indexOf(currFrag.getSequence().substring(currLen
- 20)); assemblyFragments.remove(tempFrag);
break; } // Else if there is a match between the last 20 bp of currFrag and the backwards strand of tempFrag
else if
(revComp.run(tempFrag.getSequence()).contains(currFrag.getSequence().substring(currLen
- 20))) { matchedFrag = new Polynucleotide(revComp.run(tempFrag.getSequence()));
matchedHomologousRegionEndIndex = 20 +
revComp.run(tempFrag.getSequence()).indexOf(currFrag.getSequence().substring(currLen
- 20)); assemblyFragments.remove(tempFrag);
break; } } if (matchedFrag == null) { throw new
Exception("The provided assembly fragments cannot be joined together
because there are not enough homologous regions between them"); } //added
if statement to catch degenerate base pairs if (!homologyRegion.matches("[ATCG]+"))
{ throw new IllegalArgumentException("The
provided assembly contains degenerate base pairs, assembly failed. "); } currHomologousRegionStartIndex = currFrag.getSequence().length()
- matchedHomologousRegionEndIndex; String currHomologousRegion =
currFrag.getSequence().substring(currHomologousRegionStartIndex); String matchedHomologousRegion = matchedFrag.getSequence().substring(0,
matchedHomologousRegionEndIndex); if (!(currHomologousRegion.equals(matchedHomologousRegion)))
{ throw new Exception("In a Gibson assembly step, the fragment ends do not
match"); } /\* // Find start indices of homologous region on both strands currHomologousRegionStartIndex = currFrag.getSequence().length()
- 1 - 6; matchedHomologousRegionStartIndex = matchedHomologousRegionEndIndex; while (currHomologousRegionStartIndex
> 0 && matchedHomologousRegionStartIndex
> 0 && currFrag.getSequence().charAt(currHomologousRegionStartIndex)
== matchedFrag.getSequence().charAt(matchedHomologousRegionStartIndex)) { currHomologousRegionStartIndex--; matchedHomologousRegionStartIndex--;
} \*/ // Sanity Check: currHomologousRegionStartIndex
now points to the index on the current fragment that heads the // homologous
region, matchedHomologousRegionStartIndex now points
to the index on the matched fragment that heads // the homologous region, and matchedHomologousRegionEndIndex now points to the last
index (including 6 bp matching seq) // of the homologous region on the matching
strand String currFragRegion = currFrag.getSequence().substring(0,
currHomologousRegionStartIndex); String matchedFragRegion = matchedFrag.getSequence();
Polynucleotide assembledProduct = new Polynucleotide(currFragRegion.concat(matchedFragRegion));
assemblyFragments.add(assembledProduct);
} Polynucleotide linearProduct = assemblyFragments.remove(0);
String forwardStrand = linearProduct.getSequence();
String lastSixBP = forwardStrand.substring(forwardStrand.length() - 20); int firstIndex
= forwardStrand.indexOf(lastSixBP);
if (firstIndex == forwardStrand.length()
- 20 || firstIndex < 0) { throw new
Exception("Assembly product cannot be re-circularized"); }
Polynucleotide circularProduct = new Polynucleotide(forwardStrand.substring(firstIndex,
forwardStrand.length() - 20), true); return circularProduct; }

ChatGPT

Here's the equivalent code in Google Apps
Script, based on the provided Java code:

javaCopy code

function gibson(dnaSequences) { var assemblyFragments
= dnaSequences; var revComp
= new RevComp(); revComp.initiate();
while (assemblyFragments.length > 1) { var currFrag = assemblyFragments.shift();
var currLen = currFrag.length;
var homologyRegion = currFrag.substring(currLen - 20); var matchedFrag = null;
var currHomologousRegionStartIndex; var matchedHomologousRegionStartIndex; var matchedHomologousRegionEndIndex
= 0; for (var i = 0; i <
assemblyFragments.length; i++)
{ var tempFrag = assemblyFragments[i]; if (tempFrag.indexOf(homologyRegion) > -1) { matchedFrag
= tempFrag; matchedHomologousRegionEndIndex
= 20 + tempFrag.indexOf(homologyRegion);
assemblyFragments.splice(i,
1); break; } else if (revComp.run(tempFrag).indexOf(homologyRegion) > -1)
{ matchedFrag = revComp.run(tempFrag); matchedHomologousRegionEndIndex
= 20 + revComp.run(tempFrag).indexOf(homologyRegion); assemblyFragments.splice(i, 1); break;
} } if (!matchedFrag) { throw new Error("The
provided assembly fragments cannot be joined together because there are not
enough homologous regions between them"); } if (!/^[ATCG]+$/.test(homologyRegion)) { throw new Error("The provided
assembly contains degenerate base pairs, assembly failed"); } currHomologousRegionStartIndex = currFrag.length
- matchedHomologousRegionEndIndex; var currHomologousRegion = currFrag.substring(currHomologousRegionStartIndex); var matchedHomologousRegion
= matchedFrag.substring(0, matchedHomologousRegionEndIndex);
if (currHomologousRegion !== matchedHomologousRegion)
{ throw new Error("In a Gibson assembly step, the fragment ends do not
match"); } var currFragRegion = currFrag.substring(0, currHomologousRegionStartIndex);
var matchedFragRegion = matchedFrag;
var assembledProduct = currFragRegion.concat(matchedFragRegion); assemblyFragments.push(assembledProduct); } var linearProduct
= assemblyFragments.shift(); var forwardStrand
= linearProduct; var lastSixBP
= forwardStrand.substring(forwardStrand.length
- 20); var firstIndex = forwardStrand.indexOf(lastSixBP); if (firstIndex === forwardStrand.length - 20 || firstIndex
< 0) { throw new Error("Assembly product cannot be
re-circularized"); } var circularProduct = forwardStrand.substring(firstIndex,
forwardStrand.length - 20); return circularProduct

finish it

ChatGPT

I'm sorry, it looks like my previous answer
was cut off. Here's the complete code in Google Apps Script:

javaCopy code

function gibson(dnaSequences) { var assemblyFragments
= dnaSequences; var revComp
= new RevComp(); revComp.initiate();
while (assemblyFragments.length > 1) { var currFrag = assemblyFragments.shift();
var currLen = currFrag.length;
var homologyRegion = currFrag.substring(currLen - 20); var matchedFrag = null;
var currHomologousRegionStartIndex; var matchedHomologousRegionStartIndex; var matchedHomologousRegionEndIndex
= 0; for (var i = 0; i <
assemblyFragments.length; i++)
{ var tempFrag = assemblyFragments[i]; if (tempFrag.indexOf(homologyRegion) > -1) { matchedFrag
= tempFrag; matchedHomologousRegionEndIndex
= 20 + tempFrag.indexOf(homologyRegion);
assemblyFragments.splice(i,
1); break; } else if (revComp.run(tempFrag).indexOf(homologyRegion) > -1)
{ matchedFrag = revComp.run(tempFrag); matchedHomologousRegionEndIndex
= 20 + revComp.run(tempFrag).indexOf(homologyRegion); assemblyFragments.splice(i, 1); break;
} } if (!matchedFrag) { throw new Error("The
provided assembly fragments cannot be joined together because there are not
enough homologous regions between them"); } if (!/^[ATCG]+$/.test(homologyRegion)) { throw new Error("The provided
assembly contains degenerate base pairs, assembly failed"); } currHomologousRegionStartIndex = currFrag.length
- matchedHomologousRegionEndIndex; var currHomologousRegion = currFrag.substring(currHomologousRegionStartIndex); var matchedHomologousRegion
= matchedFrag.substring(0, matchedHomologousRegionEndIndex);
if (currHomologousRegion !== matchedHomologousRegion)
{ throw new Error("In a Gibson assembly step, the fragment ends do not
match"); } var currFragRegion = currFrag.substring(0, currHomologousRegionStartIndex);
var matchedFragRegion = matchedFrag;
var assembledProduct = currFragRegion.concat(matchedFragRegion); assemblyFragments.push(assembledProduct); } var linearProduct
= assemblyFragments.shift(); var forwardStrand
= linearProduct; var lastSixBP
= forwardStrand.substring(forwardStrand.length
- 20); var firstIndex = forwardStrand.indexOf(lastSixBP); if (firstIndex === forwardStrand.length - 20 || firstIndex
< 0) { throw new Error("Assembly product cannot be
re-circularized"); } var circularProduct = forwardStrand.substring(firstIndex,
forwardStrand.length -

continue from " var circularProduct
= forwardStrand.substring(firstIndex,
forwardStrand.length -"

ChatGPT

Here's the rest of the code:

kotlinCopy code

var circularProduct
= forwardStrand.substring(firstIndex,
forwardStrand.length - 20); return circularProduct; }

I'm having trouble following this code. Try to
make it more succinct. In particular, don't use these gigantic tokens for the
variables

ChatGPT

Sure, here's a more concise version of the
code:

javaCopy code

function gibson(seqs)
{ var frag = seqs; var rev = new RevComp(); rev.initiate(); while (frag.length
> 1) { var curr = frag.shift();
var homology = curr.substring(curr.length
- 20); var match = null; var currStart; var matchStart; var matchEnd = 0; for
(var i = 0; i < frag.length; i++) { var temp =
frag[i]; if (temp.indexOf(homology)
> -1) { match = temp; matchEnd = 20 + temp.indexOf(homology); frag.splice(i, 1); break; } else if (rev.run(temp).indexOf(homology) > -1) { match = rev.run(temp);
matchEnd = 20 + rev.run(temp).indexOf(homology); frag.splice(i, 1); break; } } if (!match) { throw new Error("Fragments
cannot be joined due to insufficient homology"); } if
(!/^[ATCG]+$/.test(homology)) { throw new Error("Assembly contains
degenerate base pairs, failed"); } currStart = curr.length - matchEnd; var currSeq = curr.substring(currStart); var matchSeq = match.substring(0, matchEnd); if
(currSeq !== matchSeq) { throw
new Error("Fragment ends do not match in assembly step"); } var currRegion = curr.substring(0, currStart); var matchRegion =
match; var product = currRegion.concat(matchRegion); frag.push(product);
} var linear = frag.shift(); var forward = linear; var
last = forward.substring(forward.length
- 20); var first = forward.indexOf(last); if (first
=== forward.length - 20 || first < 0) { throw new Error("Assembly
product cannot be circularized"); } var circular = forward.substring(first,
forward.length - 20); return circular; }

The reverse complement algorithm is called
"revcomp(seq)" where seq is a string with a
dna sequence,, and it returns a string dna

ChatGPT

Understood. Here's the updated code
incorporating the **revcomp** function:

scssCopy code

function gibson(seqs)
{ var frag = seqs; while (frag.length > 1) { var curr = frag.shift(); var homology
= curr.substring(curr.length
- 20); var match = null; var currStart; var matchStart; var matchEnd = 0; for
(var i = 0; i < frag.length; i++) { var temp =
frag[i]; if (temp.indexOf(homology)
> -1) { match = temp; matchEnd = 20 + temp.indexOf(homology); frag.splice(i, 1); break; } else if (revcomp(temp).indexOf(homology) > -1) { match = revcomp(temp);
matchEnd = 20 + revcomp(temp).indexOf(homology); frag.splice(i, 1); break; } } if (!match) { throw new Error("Fragments
cannot be joined due to insufficient homology"); } if
(!/^[ATCG]+$/.test(homology)) { throw new Error("Assembly contains
degenerate base pairs, failed"); } currStart = curr.length - matchEnd; var currSeq = curr.substring(currStart); var matchSeq = match.substring(0, matchEnd); if
(currSeq !== matchSeq) {
throw new Error("Fragment ends do not match in assembly step"); } var
currRegion = curr.substring(0,
currStart); var matchRegion
= match; var product = currRegion.concat(matchRegion); frag.push(product);
} var linear = frag.shift(); var forward = linear; var
last = forward.substring(forward.length
- 20); var first = forward.indexOf(last); if (first
=== forward.length - 20 || first < 0) { throw new Error("Assembly
product cannot be circularized"); } var circular = forward.substring(first,
forward.length - 20); return circular; } function revcomp(seq) { // implementation of reverse complement
algorithm }

I have some errors. What are the data types of
" var frag = seqs; while (frag.length > 1) {
var curr = frag.shift();"

ChatGPT

In the code **var frag = seqs**, **seqs**
is an array of strings representing DNA sequences and **frag** is a
reference to the same array.

In the while loop, **frag.length**
returns the number of elements in the **frag** array. **frag.shift****()**
removes the first element of the **frag** array and returns it, so **curr** is a string representing a DNA sequence.

I'm getting: Error TypeError:
curr.substring is not a function (line 183).

ChatGPT

The error is indicating that **curr.substring** is not a function. This error is
likely occurring because **curr** is not a string
but some other type of object.

Make sure that **curr**
is indeed a string by adding a check before using the **substring** method:

javascriptCopy code

if (typeof curr !== 'string') { throw new Error('Expected curr to be a string, got ' + typeof
curr); }

I added that, and got: Error Error: Expected curr to be a
string, got object (line 184).

ChatGPT

It seems that **curr**
is an object, not a string. This could be due to an error in the implementation
of the **shift** method or the **seqs** array itself not being an array
of strings.

You could add a check to make sure that **seqs**
is indeed an array of strings:

javascriptCopy code

if (!Array.isArray(seqs)
|| !seqs.every(function (el)
{ return typeof el === 'string';
})) { throw new Error('Expected seqs to be an array of strings'); }

This check should be added before the **var
frag = seqs** line to ensure that the input is valid.

I added that, and got: Error Error: Expected seqs to be an array of strings (line 180).

ChatGPT

It seems that **seqs** is not an array of
strings as expected. You should check the input to make sure it's an array of
strings and modify it if necessary before using it in the function.

If you need help with the modification, please
provide more context about the input format and what needs to be done to make
it an array of strings.

reduce this function gibson
down to pseudocode. Exclude all the exception scenarios, just outline the main
logic at a high level

ChatGPT

Here's the high-level pseudocode for the **gibson** function:

scssCopy code

function gibson(seqs)
{ var frag = seqs; while (frag.length > 1) { var curr = frag.shift(); var homology
= curr[curr.length - 20, curr.length]; var match = null; for (var i = 0; i < frag.length;
i++) { var temp = frag[i];
if (temp.includes(homology)) { match = temp; frag.splice(i, 1); break; } else
if (revcomp(temp).includes(homology)) { match = revcomp(temp); frag.splice(i, 1); break; } } if (!match) { break; } var currRegion = curr[0, curr.length - 20]; var matchRegion
= match; var product = currRegion.concat(matchRegion); frag.push(product);
} return frag[0]; } function revcomp(seq) { //
implementation of reverse complement algorithm }

by pseudocode I mean give it in more plain english

ChatGPT

Here's the logic of the **gibson**
function in plain English:

1.  
Create a variable **frag** that refers to
the input **seqs** array.

2.  
Repeat the following steps until there's only
1 element left in the **frag** array:

3.  
Remove the first element from **frag** and
assign it to a variable **curr**.

4.  
Get the last 20 characters of **curr** and assign it to a variable **homology**.

5.  
Set a variable **match** to **null**.

6.  
Loop through each element in the **frag**
array: 1. If the current element includes **homology**, set **match** to
that element and remove it from **frag**. 2. If the reverse complement of
the current element includes **homology**, set **match** to the reverse
complement and remove it from **frag**. 3. If a match is found, break out of
the loop.

7.  
If no match was found, exit the outer loop.

8.  
Concatenate the first **curr.length****- 20** characters of **curr** and **match**
and add the result to the end of **frag**.

9.  
Return the first element of **frag**.

Note: The **revcomp**
function returns the reverse complement of a DNA sequence.

I'm pretty sure you have over complicated
this. Try to simplify it.

ChatGPT

Here's a simplified version of the **gibson** function in plain English:

1.  
Create a variable **result** that refers to
the first element of the input **seqs** array.

2.  
Repeat the following steps until there's only
1 element left in the **seqs** array:

3.  
Remove the first element from **seqs** and
assign it to a variable **curr**.

4.  
Get the last 20 characters of **curr** and assign it to a variable **homology**.

5.  
Loop through each element in the **seqs**
array: 1. If the current element includes **homology**, set **curr** to the concatenation of **curr**
(without the last 20 characters) and the current element, and remove the
current element from **seqs**. 2. If a match is found, break out of the
loop.

6.  
If no match was found, set **result** to **curr** and exit the outer loop.

7.  
Return **result**.

Rewrite the function according to this new
pseudocode

ChatGPT

Here's an implementation of the **gibson** function based on the updated pseudocode:

scssCopy code

function gibson(seqs)
{ var result = seqs[0]; while (seqs.length > 1) { var
curr = seqs.shift(); var
homology = curr.substring(curr.length
- 20); for (var i = 0; i
< seqs.length; i++) { if
(seqs[i].includes(homology)) { curr
= curr.substring(0, curr.length
- 20) + seqs[i]; seqs.splice(i, 1); break; } } if (seqs.length
=== 1) { result = curr; break; } } return result; }

Top of Form

 

Bottom of Form
