# Supplementary material for: Enabling AI in synthetic biology through Construction File specification
Source: PLoS One. 2023 Nov 13;18(11):e0294469. doi: 10.1371/journal.pone.0294469 (PMC10642840; doi:10.1371/journal.pone.0294469)
Supplement: S1 File — This ZIP file contains all essential supporting documents, including specification documents, chats, movies, Construction Files, and sequences, as referenced in the main text. A detailed description of contents is provided in the accompanying README.txt. (ZIP) [file pone.0294469.s001.zip › S1_CF_Supplementa/Chats/pydna_plugin_test.html]

PyDNA\_CF\_Simulator plugin test - A ShareGPT conversation 

Model: Plugins
•
Enabled plugins:

simulate this exactly as is:
# Define sequences
forward CCGCAACACACTTAACCTTG
reverse GTGGTTGTGGCCGGTCAAATC
template CCGCAACACACTTAACCTTGGCGTCGGGATACGTACATTGGAGAACGGTTGGCTGTACGGACTTAATACTTTTTATGATAATGATTTGACCGGCCACAACCACCG
# Define steps
PCR forward reverse template product

The simulation of your PCR process was successful. Here are the details of the sequences involved:

1. Forward Primer:

   - Sequence: CCGCAACACACTTAACCTTG
   - 5' Modification: Hydroxyl
   - 3' Modification: None
   - Is Circular: No
   - Is Double Stranded: No
2. Reverse Primer:

   - Sequence: GTGGTTGTGGCCGGTCAAATC
   - 5' Modification: Hydroxyl
   - 3' Modification: None
   - Is Circular: No
   - Is Double Stranded: No
3. Template:

   - Sequence: CCGCAACACACTTAACCTTGGCGTCGGGATACGTACATTGGAGAACGGTTGGCTGTACGGACTTAATACTTTTTATGATAATGATTTGACCGGCCACAACCACCG
   - 5' Modification: None
   - 3' Modification: None
   - Is Circular: Yes
   - Is Double Stranded: Yes
4. Product:

   - Sequence: CCGCAACACACTTAACCTTGGCGTCGGGATACGTACATTGGAGAACGGTTGGCTGTACGGACTTAATACTTTTTATGATAATGATTTGACCGGCCACAACCAC
   - 5' Modification: Hydroxyl
   - 3' Modification: Hydroxyl
   - Is Circular: No
   - Is Double Stranded: Yes

Please let me know if you need further assistance.
